# Comparative In Vitro Study of the Cytotoxic Effects of Doxorubicin’s Main Metabolites on Cardiac AC16 Cells Versus the Parent Drug

**DOI:** 10.1007/s12012-024-09829-6

**Published:** 2024-02-13

**Authors:** Ana Reis-Mendes, Cláudia Vitorino-Oliveira, Mariana Ferreira, Félix Carvalho, Fernando Remião, Emília Sousa, Maria de Lourdes Bastos, Vera Marisa Costa

**Affiliations:** 1 Associate Laboratory i4HB - Institute for Health and Bioeconomy, Faculty of Pharmacy, University of Porto, 4050-313 Porto, Portugal; 2 Laboratory of Toxicology, Department of Biological Sciences, Faculty of Pharmacy, UCIBIO - Applied Molecular Biosciences Unit, University of Porto, 4050-313 Porto, Portugal; 3 Laboratory of Organic and Pharmaceutical Chemistry, Chemistry Department, Faculty of Pharmacy, University of Porto, 4050-313 Porto, Portugal; 4 CIIMAR - Interdisciplinary Centre of Marine and Environmental Research, 4450‐208 Porto, Portugal; 5 Toxicology Laboratory, Faculty of Pharmacy, UCIBIO, University Porto, Rua de Jorge Viterbo Ferreira, 228, 4050-313 Porto, Portugal

**Keywords:** Doxorubicin, AC16 cardiac cells, Biotransformation, Doxorubicinol, Cardio-oncology, Anticancer therapy, Cardiotoxicity

## Abstract

Doxorubicin (DOX; also known as adriamycin) serves as a crucial antineoplastic agent in cancer treatment; however, its clinical utility is hampered by its’ intrinsic cardiotoxicity. Although most DOX biotransformation occurs in the liver, a comprehensive understanding of the impact of DOX biotransformation and its’ metabolites on its induced cardiotoxicity remains to be fully elucidated. This study aimed to explore the role of biotransformation and DOX's main metabolites in its induced cardiotoxicity in human differentiated cardiac AC16 cells. A key discovery from our study is that modulating metabolism had minimal effects on DOX-induced cytotoxicity: even so, metyrapone (a non-specific inhibitor of cytochrome P450) increased DOX-induced cytotoxicity at 2 µM, while diallyl sulphide (a CYP2E1 inhibitor) decreased the 1 µM DOX-triggered cytotoxicity. Then, the toxicity of the main DOX metabolites, doxorubicinol [(DOXol, 0.5 to 10 µM), doxorubicinone (DOXone, 1 to 10 µM), and 7-deoxydoxorubicinone (7-DeoxyDOX, 1 to 10 µM)] was compared to DOX (0.5 to 10 µM) following a 48-h exposure. All metabolites evaluated, DOXol, DOXone, and 7-DeoxyDOX caused mitochondrial dysfunction in differentiated AC16 cells, but only at 2 µM. In contrast, DOX elicited comparable cytotoxicity, but at half the concentration. Similarly, all metabolites, except 7-DeoxyDOX impacted on lysosomal ability to uptake neutral red. Therefore, the present study showed that the modulation of DOX metabolism demonstrated minimal impact on its cytotoxicity, with the main metabolites exhibiting lower toxicity to AC16 cardiac cells compared to DOX. In conclusion, our findings suggest that metabolism may not be a pivotal factor in mediating DOX's cardiotoxic effects.

**Graphical Abstract:**

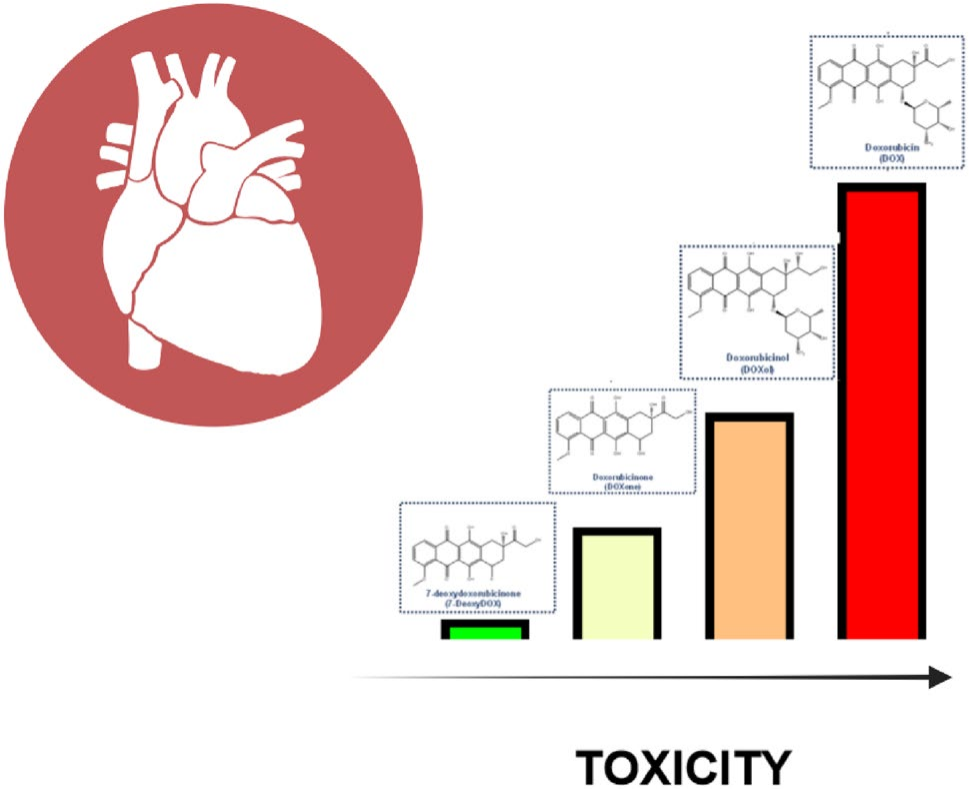

## Introduction

Doxorubicin (DOX, also known as adriamycin) is used as adjuvant therapy for breast cancer, and it is employed in the treatment of over ten other cancers. It is usually given as an intravenous injection at doses ranging from 40 mg/m^2^ to 75 mg/m^2^ every 21 days [1]. This is possibly the most frequently used drug against cancer, as a single agent, but it is mostly used in combination therapy. DOX is a topoisomerase II inhibitor and DOX’s ability to intercalate into the DNA helix and/or bind to DNA replication and transcription proteins causes the inhibition of DNA replication and synthesis [2]. Its high curative efficacy comes with an added risk of serious adverse effects, with cardiotoxicity being life threatening. Pericarditis, myocarditis, arrhythmias, and acute left ventricular failure that can lead to heart failure are known cardiotoxic features that come with DOX clinical use. The likelihood of developing cardiomyopathy is most commonly associated with a lifelong cumulative dose of DOX. In addition, there is a recognized additive or synergistic risk for cardiomyopathy in patients who have undergone radiotherapy to the mediastinum or received concurrent therapy with other known cardiotoxic agents [1].

Regarding DOX pharmacokinetics, it has a high distribution volume, with high accumulation at the cellular level [3]. Tissues obtained from autopsies of 35 patients who had been administered DOX before death in different time points of their lives, revealed that the heart exhibited the fourth-highest levels of DOX. The patients had received doses ranging from 30 to 670 mg/m^2^, and the interval since the last treatment varied between 1 and 931 days [4], showing DOX long permanence in the heart. In mice with tumours, after administering a single dose of 10 mg/kg DOX, the animal hearts exhibited the highest relative total tissue concentrations of DOX after 24 h [5].

**Fig. 1:**
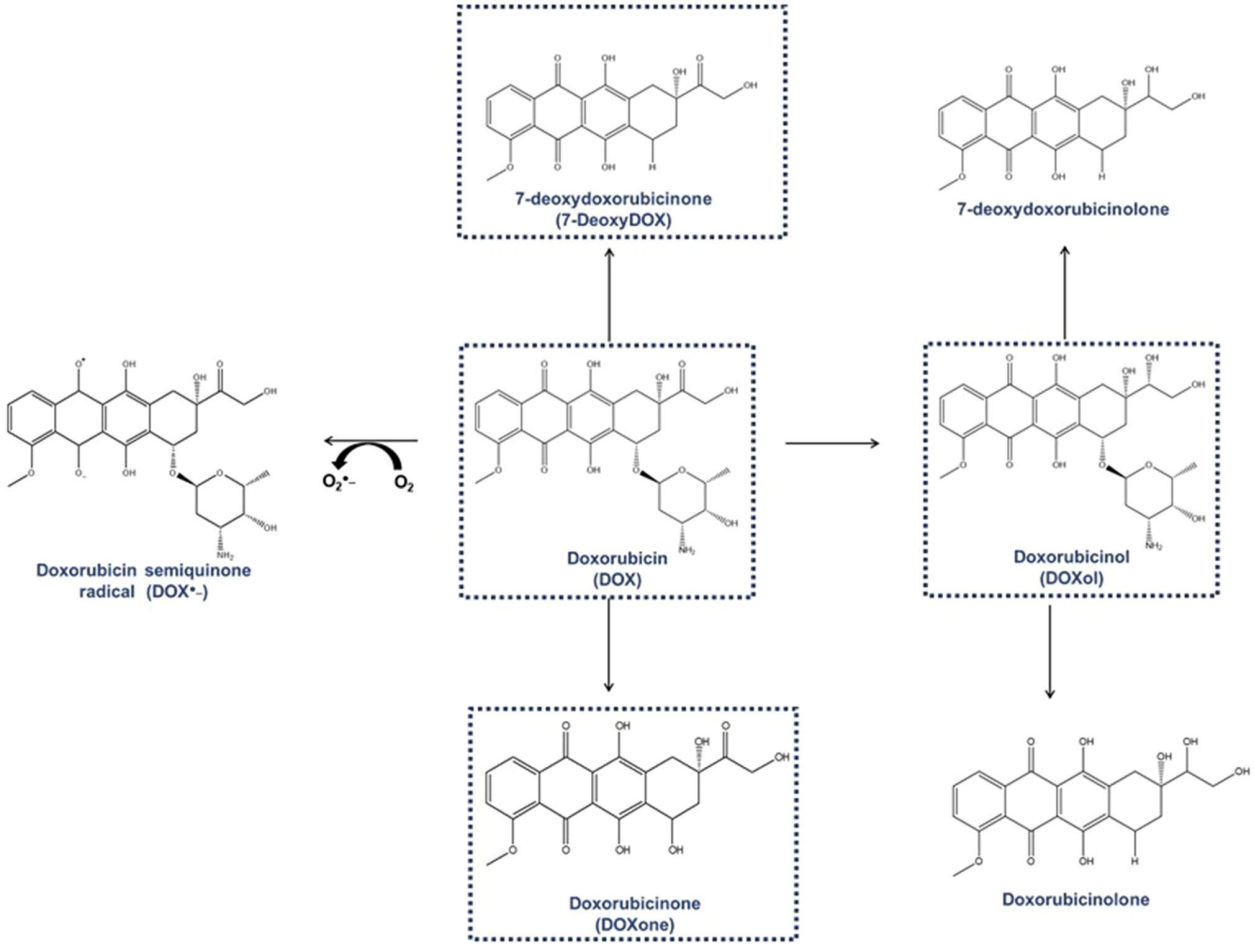
Metabolites of doxorubicin (DOX), including doxorubicinol (DOXol), doxorubicinone (DOXone), 7-deoxydoxorubicinone (7-deoxyDOX), 7-deoxydoxorubicinolone, and doxorubicinolone

Despite its large tissue accumulation, DOX also suffers extensive biotransformation, primarily within the hepatic system. DOX's most common metabolization step in humans occurs through nicotinamide-adenine dinucleotide reduced form (NADH)-dependent two-electron reduction of its carbonyl side chain, forming a secondary alcohol called doxorubicinol (DOXol) [6] (Fig. 1). This reduction step is catalysed by aldo/keto reductases or carbonyl reductases [7, 8]. Regarding the previously mentioned study by Stewart and co-workers on *post-mortem* samples, the heart ranked 5th on DOXol accumulation [4], which shows the large cardiac permanence of both the parent drug and this metabolite. DOXol is more hydrophilic than DOX, which favours its retention within the cardiomyocytes after DOX metabolism [3, 4, 9, 10]. Finally, the working hypothesis that DOXol can contribute to DOX cardiotoxicity has been placed for a long time [11, 12], and we also made research in the past to test that hypothesis [13]. In addition, DOX (and also DOXol) can undergo a minor metabolic pathway called deglycosydation, which occurs after the breakage of the glycosidic bond, producing aglycones with higher lipophilicity [3, 10]. The aglycones doxorubicinone [or 7-hydroxydoxorubicin aglycone (DOXone)), and 7-deoxydoxorubicinone [or 7-deoxydoxorubicin aglycone (7-DeoxyDOX)] can be derived from DOX by the reductive cleavage of glycosidic bond and carbonyl side-chain group by NADPH-dependent hydrolase and reductase-type glycosidases, respectively [9]. Similarly, the formation of aglycones from DOXol is achieved by reductive removal of the C7-linked daunosamine sugar group via a semiquinone intermediate and subsequent protonation of the C7-aglycone radical to form 7-deoxydoxorubicinolone (or 7-deoxydoxorubicinol aglycone), or starting on DOXol by acid-catalysed hydrolysis of the glycosidic bond, releasing the sugar component to form doxorubicinolone (or 7-hydroxydoxorubicinol aglycone) [9]. On the other hand, DOX can suffer a one-electron reduction of the quinone ring mainly by cytochrome P450 reductase, NADH dehydrogenase, or nicotinamide-adenine dinucleotide phosphate (NADPH) oxidases to form a semiquinone-free radical (DOX^•−^). Intracellularly DOX accumulates in the mitochondria, being that accumulation facilitated by its high affinity for cardiolipin. As a result, mitochondria commonly serve as a site for this reduction process. The radical of DOX is unstable and can rapidly regenerate back to the parent quinone by reducing O_2_ to O_2_^•−^ (radical anion superoxide) [14–17] (Fig. 1). This route has been by far the most studied related pathway to explain cardiotoxicity via oxidative stress; however, none of the clinical studies involving the administration of anti-oxidants had any particular protective effect so far and no current hypothesis explains entirely DOX-induced cardiotoxicity [18, 19]. Other routes of biotransformation have been less explored regarding DOX-inherent cardiotoxicity. Moreover, the studies undergone focusing on the putative role of DOX metabolites on its induced toxicity, usually evaluate the effect of one single metabolite or the detection of some metabolites [9, 10, 20, 21], being that a broader and comparative study is lacking. Therefore, we aimed, in the present work, to test all commercially available DOX metabolites, at clinically relevant concentrations, and compare their toxicity with that of the parent drug. We investigated deeper and evaluated the impact of several metabolic and autophagy modulators on the cytotoxicity elicited by DOX to the human cardiomyocyte cell line AC16, to determine the potential involvement of some metabolic pathways in the potential cardiotoxic effects of this blockbuster anticancer drug.

## Materials and Methods

### Cell Culture Materials and Chemicals

AC16 human cardiomyocytes cell line was obtained from Sigma-Aldrich (St. Louis, MO, USA). DOX hydrochloride (ab120629) was obtained by Abcam (Cambridge, UK). Doxorubicinol HCl (DOXol) was purchased to MeD CHEM101 (Plymouth Meeting, Pennsylvania). Doxorubicinone (DOXone) and 7-deoxydoxorubicin aglycone (7-DeoxyDOX) were acquired by Santa Cruz Biotechnology (Heidelberg, Germany). Neutral red (NR) solution, sodium bicarbonate, gelatine from bovine skin, trypan blue solution 0.4% (*w*/*v*), trypsin/ethylenediaminetetraacetic acid solution, acridine orange, ethidium bromide, bovine serum albumin, dimethyl sulphoxide (DMSO), 3-methyladenine (3-MA), diallyl sulphide (DAS), metyrapone (MTP), 1-aminobenzotriazole (1-ABT), carbonyl cyanide 3-chlorophenylhydrazone (CCCP), and phenobarbital (PHB) were obtained from Sigma-Aldrich (Germany). 3-(4,5-Dimethylthiazol-2-yl)-2,5-diphenyl tetrazolium bromide (MTT) was purchased to Alfa Aesar (Massachusetts, United States), and fibronectin from bovine plasma was purchased from Thermo Fisher Scientific (Kandel, Germany). Phosphate-buffered saline (PBS), 1% penicillin, 100 µg/mL streptomycin, and Hank’s balanced salt solution were purchased from Biochrom (Berlin, Germany). Dulbecco´s Modified Eagle´s Medium/Nutrient F12 Ham (DMEM/F12) powder, horse serum (HS) heat inactivated, and foetal bovine serum (FBS) heat inactivated were obtained from Alfagene (Carcavelos, Portugal). All plastic sterile material used in cell culture was obtained from Corning Star (Corning, NY, USA).

### Cell Culture Proceedings

The toxicological evaluation of DOX and its metabolites (DOXol, DOXone, and 7-DeoxyDOX) was carried out in the human cardiomyocyte cell line AC16. The AC16 cells are a cardiac model and are derived through the fusion of SV40 transformed, uridine auxotroph human fibroblasts, devoid of mitochondrial DNA with human ventricular cardiomyocytes [22]. The fused cells have cardiomyocyte-specific markers and retain nuclear and mitochondrial DNA from the primary cardiomyocytes [22]. AC16 cells were maintained in DMEM/F12 medium (supplemented with 12.5% FBS, 1% penicillin, and 100 µg/mL streptomycin) at 37 °C in a 5% CO_2_ humidified atmosphere [24]. Cell passaging was done by trypsinization, and the cells were used no more than tenth passages after thawing [22]. When the cell population reached approximately 90% confluence, experiments were performed. AC16 cells were seeded at a density of 32.5 × 10^4^ cells/cm^2^ in coated surfaces with 12.5 µg/mL fibronectin in 0.02% gelatine, for at least 1 h at 37 °C [23]. After seeding, AC16 cells were incubated for 24 h to allow cellular adherence. Then, two different protocols were used: (1) proliferative cells were exposed to the drugs while maintained in a proliferative medium (DMEM/F12 medium supplemented with 12.5% FBS, 1% antibiotics) for a maximum of 72 h; or (2) the differentiation procedure began 24 h after seeding when AC16 cells were exposed to a differentiation medium (DMEM/F12 medium supplemented with 2% HS and 1% antibiotics) for 24 h. Drug incubation in cells in the differentiated state never surpassed 48 h, as it was reported that cell death would prevail [22].

DOX, 7-DeoxyDOX, DOXone, and DOXol were prepared in sterile PBS without Ca^2+^ and Mg^2+^ and stored at -20 °C. None of the compounds under testing were subjected to more than three cycles of freezing/thawing.

### Cytotoxicity Tests

For the cytotoxicity assays (MTT reduction and NR uptake assays), AC16 cells were seeded in 48-well plates and exposed to DOX (0.5 to 10 µM) for 24, 48, or 72 h in proliferative cells and 24 or 48 h in cells in the differentiated state. Another set of assays (MTT reduction and NR uptake) was performed on differentiated AC16 cells, pre-incubated with metabolism modulators: (1) MTP (0.5 mM), an inhibitor of steroid 11-β hydroxylase (CYP11B1) [24], CYP3A4 [25], and cytochrome P450-mediated ω/ω-1 hydroxylase activity [26]; (2) 1-ABT (0.5 mM), a CYP450 inhibitor [27]; (3) DAS (50 µM), a CYP2E1 inhibitor [28]; (4) PHB (1 mM), a CYP2B6 inducer and also aldo/keto reductases inhibitor [29–31]. All modulators were given to the AC16 cells for 1 h at 37 °C before DOX. The inhibitor of autophagy, 3-MA (2.5 mM) [32] was also given for 1 h at 37 °C before the addition of DOX, and its effect was assessed by the MTT reduction and the NR uptake assays. The metabolites had their cytotoxicity only evaluated on AC16 cells in the differentiated state using the later time point, 48 h. The concentrations used for DOX and DOXol are clinically relevant and are within the range of plasma concentrations found in treated cancer patients undergoing DOX therapy [33].

#### MTT Reduction Assay

The MTT colorimetric assay is based on the reduction of the tetrazolium salt to formazans by dehydrogenases, and it was performed as previously described by our group [23, 34] after a 48-h incubation with parent drug/metabolic modulators or metabolites. The MTT reduction results were expressed in the percentage of control cells (set for 100%).

#### NR Uptake Assay

NR uptake assay is based on the ability of viable cells to integrate and bind the supravital dye NR in the lysosomes [35]. The procedure was performed as previously described within the research group [23, 34]. After the cells’ incubation for 48 h with DOX, metabolic modulators, or DOX main metabolites, NR uptake was evaluated. The values of control cells were set to 100%, and the values in cells exposed to drugs were expressed as a percentage of control cells.

### Evaluation of Cell’s Morphology

Cellular morphology after incubation with anticancer drugs was done using phase contrast microscopy. The photographs were taken using a Nikon Eclipse TS100 inverted microscope equipped with a DS-fi1 camera (Japan). Differentiated AC16 cells were incubated for 48 h with two different concentrations (1 and 2 μM) of DOX, DOXol, DOXone, 7-DeoxyDOX, and the respective vehicle at the highest concentration used (DMSO 0.004%, for DOXone and 7-DeoxyDOX).

### Evaluation of Mitochondrial Membrane Potential

For the evaluation of mitochondrial membrane potential, a lipophilic cationic dye, JC-1 was used. The JC-1 dye is a selective mitochondrial dye that spontaneously forms J-aggregates with intense red fluorescence in healthy mitochondria. During mitochondrial membrane depolarization, the JC-1 dye reversibly changes colour from red to green [36, 37]. In this assay, AC16 cells were incubated for 48 h with the DOX (1 µM) or its metabolites (1 and 2 µM). Then, a JC-1 probe was used for each well (20 µM final concentration) and incubated for 15 min at 37 °C. After two washing steps, two fluorescent readings were done: red was read at a λ excitation maximum = 535 nm and a λ emission maximum = 595 nm, and green was read at a λ excitation maximum = 485 nm and a λ emission maximum = 535 nm in a multi-well plate reader (Biotech Synergy HT (Winooski, VT, USA)). The ratio of red and green fluorescence was calculated for each condition and mean control values were set to 100%. In parallel, a positive control for mitochondrial membrane depolarization was used: the protonophore, CCCP.

### Statistical Analysis

Results are expressed as mean ± standard deviation (SD). The outliers were identified using the ROUT method (Q = 1%), and then statistical analysis was performed. To assess data normality, the D’Agostino & Pearson normality test was performed. When using different concentrations at time points, a two-way ANOVA test was performed, followed by Tukey’s post hoc test. When using a one-time point and if results were normal, a one-way ANOVA test was performed, followed by Tukey’s post hoc test. The Kruskal–Wallis test was performed when the data did not follow a normal distribution, followed by Dunn’s post hoc test. Statistical significance was considered when *p* values < 0.05. To perform the statistical analysis, the GraphPad Prism software (version 8.4.2) (San Diego, CA, USA) was used. Details of statistical analysis can be found in the figure’s legends.

## Results

### In Proliferative AC16 Cells, DOX Produced a Time- and Concentration-Dependent Mitochondrial and Lysosome Dysfunction

**Fig. 2:**
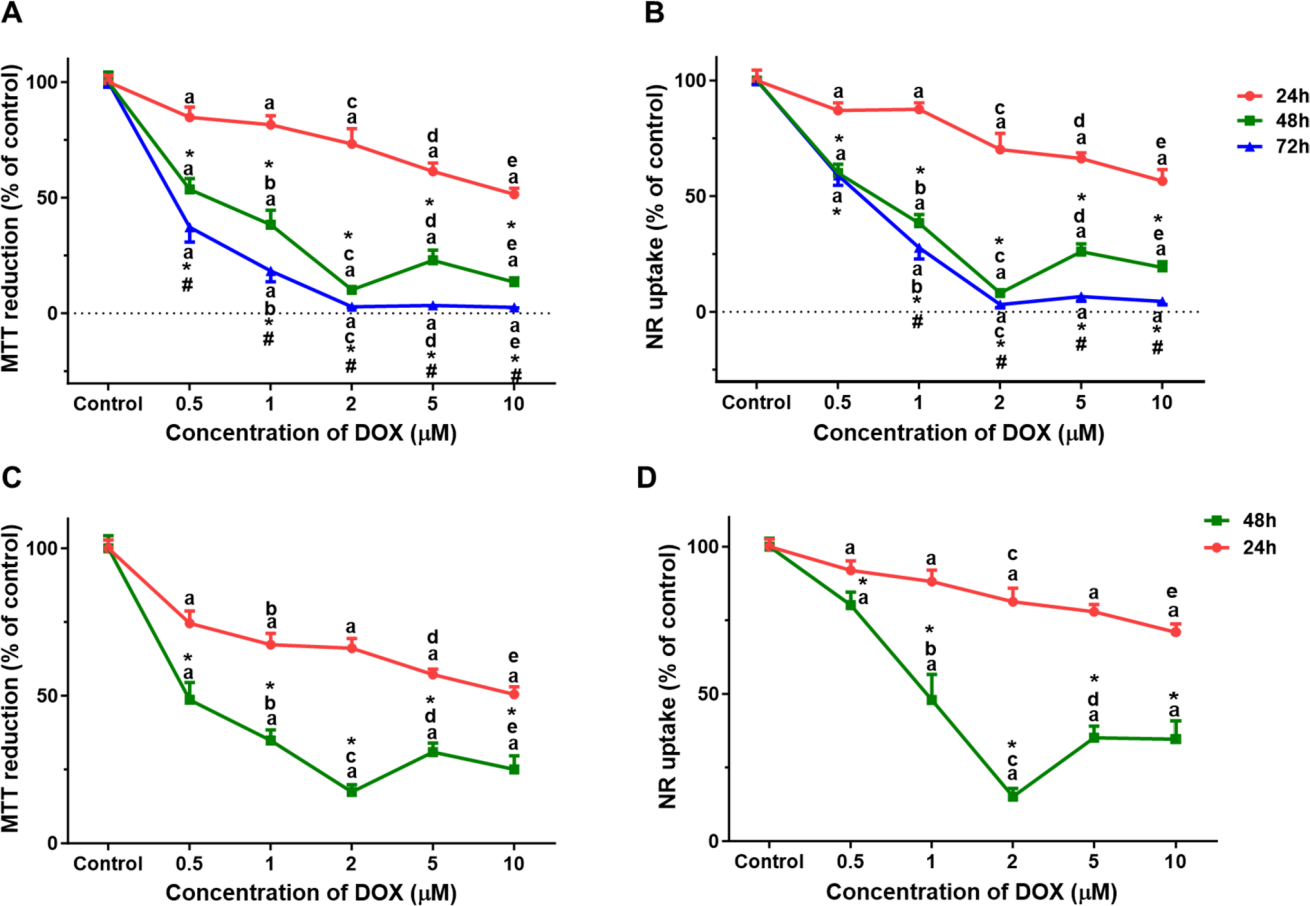
Mitochondrial and lysosomal dysfunction was evaluated by (**A**, **C**) the MTT reduction (**B**, **D**) and the NR uptake assays, respectively. **A**, **B** Proliferative AC16 cells were incubated with 0.5, 1, 2, 5, or 10 μM of DOX for 24, 48, and 72 h. **C**, **D** Differentiated AC16 cells were incubated with 0.5, 1, 2, 5, or 10 μM of DOX for 24 and 48 h. Results are presented as mean ± SD of 3–4 independent experiments (total of 12–24 wells). Statistical analyses were performed using the two-way ANOVA test, followed by Tukey’s post hoc test: (^a^*p* < 0.05 *versus* control; ^b^*p* < 0.05 *versus* 0.5 µM; ^c^*p* < 0.05 *versus* 1 µM; ^d^*p* < 0.05 *versus* 2 µM; ^e^*p* < 0.05 *versus* 5 µM; in the same time point) and (*p < 0.05 *versus* 24 h and ^#^*p* < 0.05 *versus* 48 h; in the same concentration)

The proliferative AC16 cells were directly exposed to different concentrations (0.5 to 10 µM) of DOX for 24, 48, and 72 h (Fig. 2A, B). The results of the MTT reduction and NR uptake assays showed that DOX caused cytotoxicity in a concentration-dependent manner. At 24 h, the values of MTT reduction and NR uptake assays were (i) at 0.5 μM: 84.75 ± 4.35% and 87.03 ± 3.27%, (ii) at 1 μM: 81.51 ± 3.87% and 87.55 ± 2.80%, (iii) at 2 μM: 73.20 ± 6.62% and 70.16 ± 6.98%, (iv) at 5 μM: 61.33 ± 3.67% and 66.22 ± 2.50%, and v) at 10 μM: 51.44 ± 2.64% and 56.49 ± 5.05%, when compared to control cells (100.00 ± 2.81% and 100.00 ± 4.43%), respectively.

At 48 h, the values of MTT reduction and NR uptake assays were (i) at 0.5 μM: 53.56 ± 4.69% and 59.99 ± 3.84%, (ii) at 1 μM: 38.32 ± 6.26% and 38.34 ± 3.67%, (iii) at 2 μM: 10.17 ± 1.70% and 8.16 ± 0.92%, (iv) at 5 μM: 22.92 ± 4.42% and 25.99 ± 3.40%, and (v) at 10 μM:13.53 ± 1.85% and 19.20 ± 2.48%, when compared to control cells (100.00 ± 4.30% and 100.00 ± 1.77%), respectively.

At 72 h, the values of MTT reduction and NR uptake assays were (i) at 0.5 μM: 37.22 ± 6.46% and 58.93 ± 4.31%, (ii) at 1 μM: 18.33 ± 6.45% and 27.70 ± 4.83%, (iii) at 2 μM: 2.78 ± 0.46% and 3.19 ± 1.28%, (iv) at 5 μM: 3.42 ± 0.34% and 6.63 ± 2.07%, and v) at 10 μM: 2.54 ± 0.19% and 4.49 ± 1.32%, when compared to control cells (100.00 ± 2.26% and 100.00 ± 1.79%), respectively.

### In Differentiated AC16 Cells, DOX Produced a Time- and Concentration-Dependent Mitochondrial and Lysosome Dysfunction

The observed cytotoxicity induced by DOX in the differentiated AC16 cells at different concentrations (0.5 to 10 µM) and time points (24 and 48 h) was higher when evaluated by the MTT reduction assay than by the NR uptake assay (Fig. 2C, D). At 24 h, the values of MTT reduction and NR uptake assays were (i) at 0.5 μM: 74.58 ± 4.15% and 91.94 ± 3.28%, (ii) at 1 μM: 67.29 ± 3.86% and 88.11 ± 3.87%, (iii) at 2 μM: 66.10 ± 3.36% and 81.30 ± 4.56%, (iv) at 5 μM: 57.23 ± 1.82% and 77.90 ± 2.42%, and (v) at 10 μM:50.46 ± 2.56% and 70.96 ± 2.80%, when compared to control cells (100.00 ± 2.78% and 100.00 ± 2.55%), respectively.

At 48 h, the values of MTT reduction and NR uptake assays were (i) at 0.5 μM: 48.60 ± 5.87% and 80.12 ± 4.41%, (ii) at 1 μM: 34.86 ± 3.56% and 47.95 ± 8.63%, (iii) at 2 μM: 17.39 ± 2.44% and 15.14 ± 2.77%, (iv) at 5 μM: 30.91 ± 3.03% and 35.11 ± 3.99%, and (v) at 10 μM: 25.05 ± 4.58% and 34.65 ± 6.25%, when compared to control cells (100.00 ± 4.21% and 100.00 ± 2.88%), respectively.

### Inhibition of Autophagy had no Impact on the Cytotoxicity of Doxorubicin

**Fig. 3:**
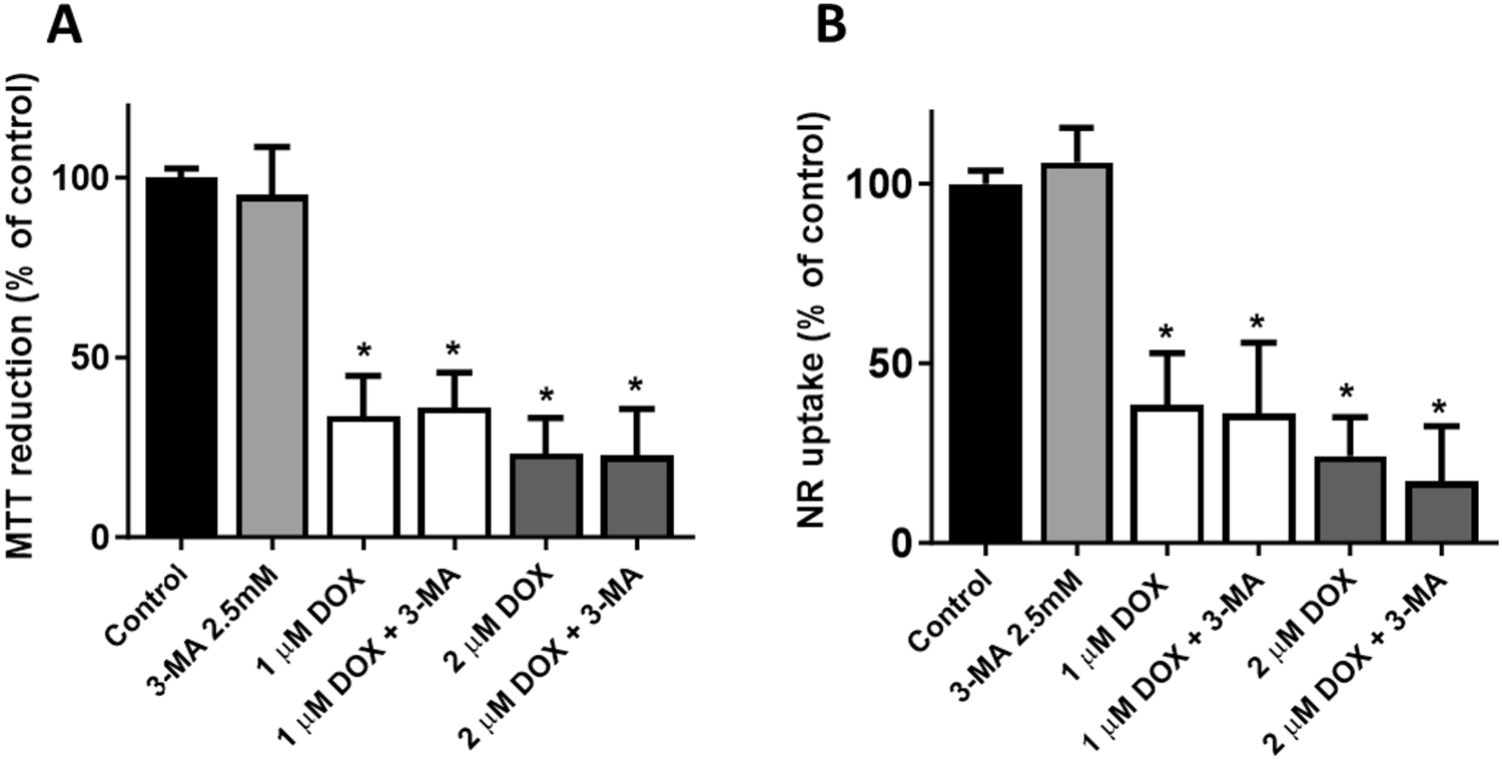
Mitochondrial and lysosomal dysfunction was evaluated by (**A**) the MTT reduction and (**B**) the NR uptake assays, respectively, in differentiated AC16 cells incubated with 1 or 2 μM of DOX for 48 h with or without: **A**, **B** 3-methyladenine (3-MA) (2.5 mM final concentration). Results are presented as mean ± SD of 4 independent experiments (total of 12–16 wells). The statistical analyses were performed using (**A**, **B**) one-way ANOVA test, followed by Tukey’s post hoc test (^*^p < 0.05 *versus* control)

3-MA is experimentally used as an inhibitor of autophagy via its inhibitory effect on class III phosphoinositide 3-kinase (PI3K-III). By interfering with the formation of phosphatidylinositol 3-phosphate, 3-MA disrupts the early stages of autophagosome formation, thereby blocking the autophagic process. At 48 h, the pre-incubation with 3-MA did not change the cytotoxicity caused by DOX (Fig. 2A, B). The incubation with 3-MA at 2.5 mM, per se did not cause any significant toxicity when compared to control cells (Fig. 3A, B). Since no differences were seen, the role of biotransformation of DOX and its impact on cytotoxicity was subsequently evaluated.

### A Small but Significant Increase in Cytotoxicity was Found in Cells Incubated with Metyrapone While Diallyl Sulphide Decreased the Cytotoxicity Caused by DOX

The differentiated AC16 cells were pre-incubated with CYP450 inhibitors (1-ABT or MTP or DAS) or CYP2B6 inducer and aldo/keto reductases inhibitor (PHB) for 1 h at 37 °C before adding DOX. The effect of the CYP450 inhibitor 1-ABT (0.5 mM) [27], of the competitive inhibitor of steroid 11-β hydroxylase (CYP11B1) MTP (0.5 mM) [38], and the CYP2E1 inhibitor DAS (50 µM) [28] in differentiated AC16 cells incubated with DOX, was evaluated through the MTT reduction and NR uptake assays at 48 h.

**Fig. 4:**
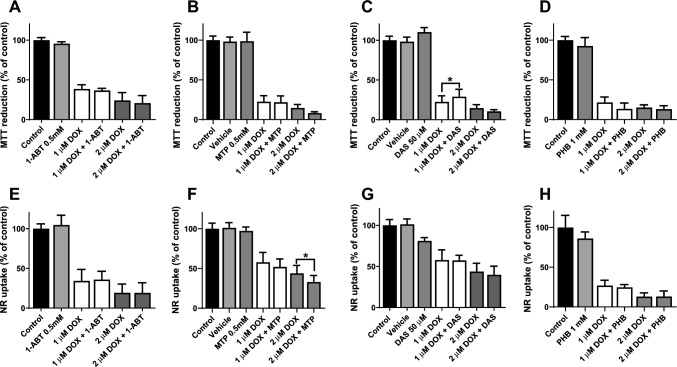
Mitochondrial and lysosomal dysfunction was evaluated by **A**, **B**, **C**, **D** the MTT reduction and **E**, **F**, **G**, **H** the NR uptake assays, respectively, in differentiated AC16 cells incubated with 1 or 2 μM of DOX for 48 h with or without: **A**, **E** 1-aminobenzotriazole (1-ABT) (0.5 mM final concentration), **B**, **F** metyrapone (MTP) (0.5 mM final concentration), **C**, **G** diallyl sulphide (DAS) (50 µM final concentration), and **D**, **H** phenobarbital (PHB) (1 mM final concentration). Results are presented as mean ± SD of 3–4 independent experiments (total of 12–16 wells). The statistical analyses were performed using **A**, **C**–**G** one-way ANOVA test, followed by Tukey’s post hoc test or [B, H] Kruskal–Wallis test, followed by Dunn’s post hoc test (*p < 0.05, 1 µM DOX *versus* 1 µM DOX + DAS) and (*p < 0.05, 2 µM DOX *versus* 2 µM DOX + MTP). DMSO (final concentration of 0.1% *v/v*) was used as the vehicle. For simplicity, the statistical data referring to meaningful changes regarding the control were omitted, as they can be found on previous graphs

At 48 h, the pre-incubation with 1-ABT, a broad CYP450 inhibitor, did not cause a significant change in mitochondrial and lysosomal dysfunction when compared to the drug alone (Fig. 4A, E). At 48 h, MTP significantly increased the cytotoxicity caused by DOX 2 µM in the NR uptake assay (Fig. 4F). However, DOX added to the cells after pre-incubation with MTP did not cause a significant change in mitochondrial dysfunction (determined by the MTT reduction assay) when compared to the drug alone (Fig. 4B). In the MTT reduction assay, DAS partially decreased the cytotoxicity induced by DOX 1 µM as can be seen in Fig. 4C. In the NR uptake assay, DAS did not change the DOX-induced cytotoxicity at 48 h (Fig. 4G).

The effects of the CYP2B6 inducer and aldo/keto reductases inhibitor [29–31], PHB, 1 mM was evaluated through the MTT reduction and NR uptake assays (Figs. 4A and C). In both assays, PHB was not able to cause significant changes in the DOX-induced cytotoxicity at 48 h (Figs. 4D, H). The incubation of 1-ABT, MTP, DAS, PHB, or DMSO (vehicle) per se did not cause any cytotoxicity when compared to control cells at either cytotoxicity test performed.

### All DOX Metabolites Tested Caused Changes in the Ability of AC16 Cells to Reduce MTT

**Fig. 5:**
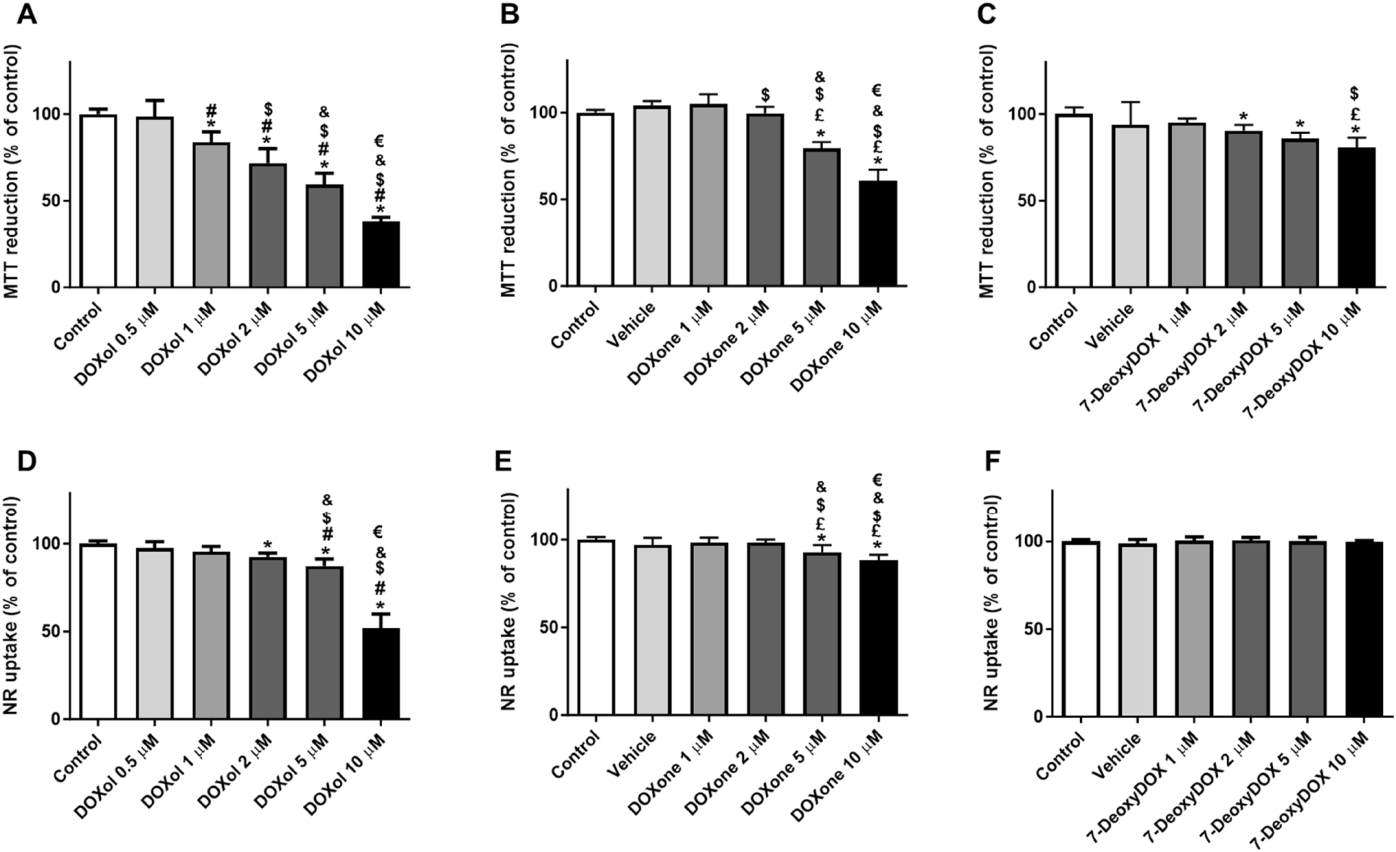
Mitochondrial and lysosomal dysfunction was evaluated by the **[**A, B, C] MTT reduction and [D, E, F] NR uptake assays, respectively. Differentiated AC16 cells incubated with **A**, **D** 0.5, 1, 2, 5, or 10 μM of doxorubicinol (DOXol), 1, 2, 5, or 10 μM **B**, **E** of doxorubicinone (DOXone) and **C**, **F** of 7-deoxydoxorubicin aglycone (7-DeoxyDOX) for 48 h. Results are presented as mean ± SD of 3 independent experiments (total of 12 wells). The statistical analyses were performed using **A**, **B**, **D**–**F** one-way ANOVA test, followed by Tukey’s post hoc test or **[C]** Kruskal–Wallis test, followed by Dunn’s post hoc test (*p < 0.05, *versus* control; ^#^*p* < 0.05 *versus* 0.5 µM; ^$^*p* < 0.05 *versus* 1 µM; ^&^*p* < 0.05 *versus* 2 µM; ^€^*p* < 0.05 *versus* 5 µM; ^£^*p* < 0.05 *versus* vehicle). DMSO (final concentration of 0.1% *v/v*) was used as the vehicle

We investigated the effect of 7-DeoxyDOX, DOXone, and DOXol on differentiated AC16 cells by exposing them to different concentrations (1 to 10 µM) of the metabolites for 48 h. The results showed that 7-DeoxyDOX, DOXone, and DOXol caused significant cytotoxicity in the MTT assay (Fig. 5). In the MTT reduction assay, the values of MTT reduction after DOXol incubation were, in percentage for the conditions 0.5 μM: 98.53 ± 9.47%, 1 μM: 83.85 ± 5.92%, 2 μM: 71.78 ± 8.36%, 5 μM: 59.32 ± 6.62%, and 10 μM: 37.94 ± 2.53%, when compared to control cells (100.00 ± 2.94%) (Fig. 5A). The values of MTT reduction after DOXone were, at 1 μM: 105.0 ± 5.71%, 2 μM: 99.62 ± 3.76%, 5 μM: 79.20 ± 3.89%, and 10 μM: 60.57 ± 6.59%, when compared to control cells (100.00 ± 1.66%) and vehicle (104.2 ± 2.54%) (Fig. 5B). The values MTT reduction after 7-DeoxyDOX were, at 1 μM: 95.02 ± 2.40%, 2 μM: 90.37 ± 3.38%, 5 μM: 85.79 ± 3.41%, and 10 μM: 80.50 ± 5.94%, when compared to control cells (100.00 ± 3.87%) and vehicle (93.80 ± 13.17%) (Fig. 5C).

Regarding the NR uptake assay, only DOXol and DOXone caused significant cytotoxicity, when compared to control cells. The values of NR uptake on AC16 cells after DOXol were, at 0.5 μM: 97.47 ± 3.70%, 1 μM: 95.44 ± 3.02%, 2 μM: 92.43 ± 2.18%, 5 μM: 87.27 ± 4.05%, and 10 μM: 51.88 ± 8.05%, when compared to control cells (100.00 ± 1.55%) (Fig. 5D). The values of NR uptake on AC16 cells after DOXone were, at 1 μM: 98.37 ± 2.77%, 2 μM: 98.36 ± 1.66%, 5 μM: 92.83 ± 4.12%, and 10 μM: 88.32 ± 3.04%, when compared to control cells (100.00 ± 1.43%) and vehicle (97.00 ± 4.05%) (Fig. 5E).

The highest concentration of DMSO (vehicle) used was tested to assess its potential toxicity. No significant differences were observed when compared to control cells, in either assay.

### DOX Caused Significant Changes in the Morphology of Differentiated AC16 Cells

**Fig. 6:**
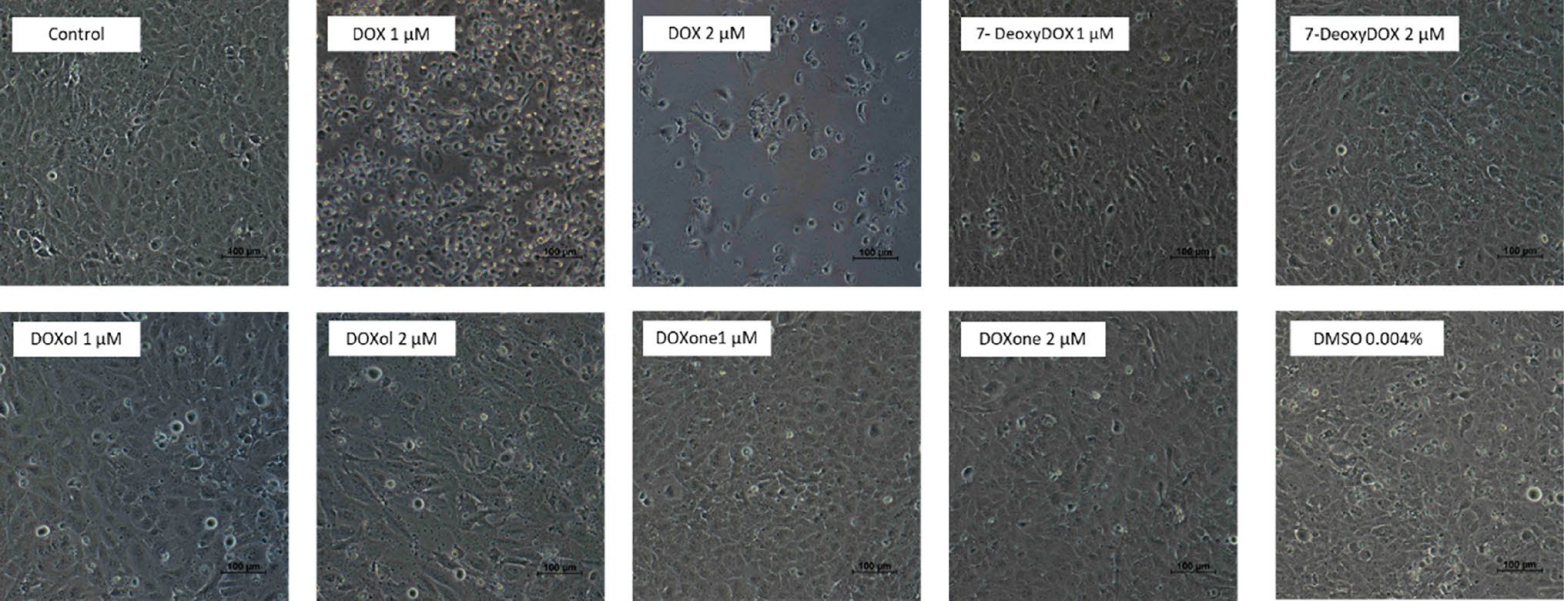
Cells’ morphology was evaluated by phase contrast microscopy. Differentiated AC16 cells were exposed for 48 h to DOX and its’ main metabolites (DOXol, DOXone, 7-DeoxyDOX), as well as to the highest concentration of vehicle used (DMSO 0.004%). The images were representative of 2 independent experiments. Scale bar: 100 μM

The incubation with DOX caused substantial alterations in cells’ morphology and cellular density (Fig. 6). Round and detached cells were evident in both concentrations, being those changes evident in the cells incubated with the higher concentration tested (2 μM). DOXol (1 and 2 μM) also induced alterations in the morphology of differentiated AC16 cells, mainly for the highest concentration tested, where cells look larger and flat. The remaining tested metabolites did not cause significant changes in cells’ morphology when compared to the control differentiated AC16 cells. No significant alterations were observed in the cells incubated with DMSO.

### DOX and the Highest Tested Concentration of all DOX Metabolites Caused Significant Changes in Mitochondrial Membrane Potential

**Fig. 7:**
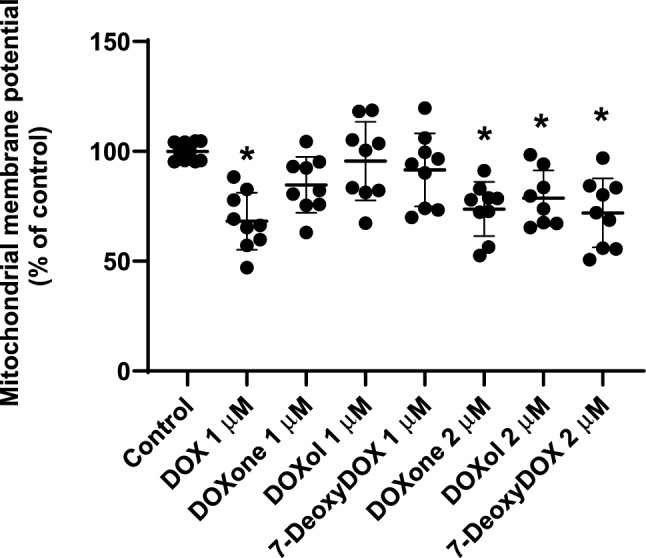
Mitochondrial membrane potential was evaluated using the JC-1 probe in differentiated AC16 cells incubated with 1 μM of DOX, 1 μM or 2 μM of doxorubicinol (DOXol), of doxorubicinone (DOXone) and 7-deoxydoxorubicin aglycone (7-DeoxyDOX) for 48 h. Results are presented as mean ± SD of 3 independent experiments (performed in triplicate). The statistical analyses were performed using one-way ANOVA test, followed by the Tukey’s post hoc test (*p < 0.05 *versus* control)

Mitochondrial membrane potential can be assessed with the probe, JC-1. JC-1 is accumulated within mitochondria and responds according to the mitochondrial membrane potential. DOX caused an impressive mitochondrial membrane potential depolarization (1 µM: 68.19 ± 13.02%) compared to the values observed in control cells (100.0 ± 4.21%). DOXol (2 µM: 78.70 ± 12.63%), DOXone (2 µM: 73.73 ± 12.28%), 7-DeoxyDOX (2 µM: 71.98 ± 15.67) only caused changes in mitochondrial membrane potential at the highest concentration tested (Fig. 7).

## Discussion

The predominant literature on DOX metabolites primarily focus on their detection, the characterization of patients’ pharmacokinetics profile, or, at most, the cytotoxic effect of one metabolite [4, 5, 9, 10, 20, 21, 39]. However, some pioneer studies, mainly from the group of Giorgio Minotti [9, 10, 20] or the 1988 work by Olson and co-workers [11] brought new perspectives on the influence of DOX metabolites on the cardiotoxicity of the parent drug. However, as far as we know, no comparative study was made using the commercially available metabolites of DOX.

Currently, there is a lack of information regarding the metabolic ability of the AC16 cell line. The enzymes herein mentioned or other related to biotransformation have not been described in the original work of Davidson and colleagues that first characterized AC16 cells [22]; However, a few papers used these cells for instance to demonstrate that AC16 cells possess cytochrome P450 2J2 (CYP2J2) [40] or that the AC16 human heart cell line can be induced to express CYP1A1 mRNA, and protein after drug exposure [41] showing their ability to undertake biotransformation of drugs. Thus, in human cardiac cells, we first compared if the state of proliferation of the cells significantly influenced the cytotoxicity of DOX in two classical cytotoxicity assays, the MTT reduction and NR uptake assays. Surprisingly, in both cells’ states, DOX caused a time- and concentration-dependent cytotoxicity up to 2 µM, beyond which the cytotoxicity exhibited a slightly but significant decrease. This biphasic response towards DOX cytotoxicity was already observed in cortical neurons where the classical cytotoxicity assays were used [42]. In that study, the authors indicated that the apparent decrease in cytotoxicity was associated with varying types of cell death. They found that at lower concentrations, cell death followed an apoptotic pattern, whereas at higher concentrations, apoptosis was inhibited and necrosis became predominant [42]. We did not assess the type of cell death in the present study, but we assessed cell morphology that confirmed cellular death by DOX and we assessed the mitochondrial membrane potential usually linked to apoptosis, which will be discussed below.

Although some researchers have reported significant differences in DOX cytotoxicity based on the differentiation state of the H9c2 cell line, [43] our data did not reveal any significant differences. Consequently, we carried on our work using differentiated AC16 cells, since these have additional markers characteristic of adult cardiomyocytes [22].

Firstly, autophagy has been described as a key but controversial component of DOX-induced cardiotoxicity [44–46]. Another work of the research group with an inhibitor of topoisomerase II, mitoxantrone, showed that its cytotoxicity was impacted by autophagy in the same cell model and similar experimental protocol [34]. Therefore, we decided to determine the role of autophagy on DOX cytotoxicity. However, in this present paradigm, no significant differences were seen after 3-MA pre-incubation, and no further studies were performed.

The studies made on DOX metabolism in vitro use liver models or at best human fractions of the heart [9, 10, 20]. Human fractions are possibly the best model to take on to perform studies on the human cardiac metabolism of DOX, but first they are not quite as available as cell lines (for ethical reasons) and second the work of Minotti’s group showed that the fractions of the metabolites formed are dependent protein ratio [9], falling short in showing what truly happens in the heart under DOX exposure. Thus, we used modulators of metabolism to determine the influence of metabolism on the cytotoxicity of DOX on a human cardiac model.

1-ABT is a non-specific, but time-dependent inhibitor of cytochrome P450 (CYP) enzymes, being mostly used to assess the relative role of oxidative metabolism on the drug under study [47, 48]. We observed no significant influence of 1-ABT in DOX-induced cytotoxicity. This suggests that either none of the formed metabolites significantly contribute to DOX cardiotoxicity or putatively toxic metabolites are not formed in significant amounts to cause toxicity. We then proceeded with the characterization of the metabolism of DOX on AC16 cells using other modulators.

MTP is a cytochrome P450 inhibitor [38]. The story of MTP is not without controversy, and while now it is described as an inhibitor of CYP11B1 (steroid 11-β hydroxylase) (inhibitory concentration 50 (IC50) = 7.83 μM) [24], CYP3A4 [25] and cytochrome P450-mediated ω/ω-1 hydroxylase activity [26], it has been described in the past to have other functions [49]. In the present work, we showed that MTP increased the cytotoxicity of the highest concentration of DOX in the NR uptake assay, while 1-ABT had no effect. Because the concentration tested of MTP, 0.5 mM, was much higher than that of the recommended IC50, we must acknowledge that possibly several or even all CYP present in the system were inhibited. On the other hand, some CYP metabolic activity persists even after one-hour incubation with 1-ABT on rat hepatocytes [47], and some CYP metabolism may still pertain in our cellular model after 1-ABT and that inhibition is not complete [47]. Furthermore, the inhibition provided by MTP results from interactions with the heme group. That interaction is reported to have medium strength, being that water-mediated contacts stabilize the inhibitory complexes at least with CYP3A4 [50]. This means that although both are CYP inhibitors, we possibly have higher inhibition of CYPs in MTP condition and that may redirect the metabolism to more toxic products.

However, another aspect must not be overruled, as we took on 48 h incubations and genetic machinery may have been changed. In the literature, the incubation of 0.5 mM MTP in primary rat hepatocytes led to the accumulation of CYP1A1 and CYP1A2 mRNA (as early as 14 h) and an obvious rise in CYP1A-associated enzymatic activity [51]. Therefore, we cannot rely on heavy conclusions set on MTP data (or 1-ABT) and the slight increase in the cytotoxicity of DOX was observed on the NR uptake assay because: 1) metabolism can be a relay to other more toxic metabolites non-dependent in CYP, like DOXol or 2) this change can be a targeted increase on the higher activity of CYP1A activity that favours the formation of aglycone derived metabolites.

The last two modulators tested and largely described in the literature are DAS and PHB. DAS is a selective inhibitor of cytochrome P450 2E1 (CYP2E1) [52]. We found partial protection in cells pre-incubated with DAS when exposed to the lowest concentration of DOX. Both DAS and its metabolites have been described as inhibitors of P450 2E1-mediated p-nitrophenol hydroxylase activity [52]. In fact, one of the DAS metabolites has been described as an irreversible inhibitor of CYP2E1 [52], which can be a factor contributing to the data seen. In addition, this partially protective action has been described in H9c2 cells incubated with another anticancer drug of the same pharmacological group, mitoxantrone [53]. Interestingly, in vivo*,* DOX (single intraperitoneal injection of 20 mg/kg) inhibited hepatic *Cyp2e1* gene expression in both male and female mice just 24 h after its administration. Nonetheless, in males, the inhibition was higher (80% inhibition) than in female mice (30% inhibition), which resulted in less inflammation [54]. One can speculate that perhaps the inhibition in males can be an overall protection feature against DOX toxicity. In fact, a review showed that most papers relate that women are more prone to cardiotoxicity of DOX, although others do not report any sex differences [55]. Still, our data corroborates in vitro that CYP2E1 has a role on DOX-induced cardiotoxicity. Considering that CYP2E1 is the most important cardiac cytochrome P450 enzyme, this data should not be disregarded.

Finally, we saw no differences regarding the pre-incubation with PHB and DOX. PHB induces the activity of a wide variety of hepatic enzymes, including NADPH-cytochrome c reductase among others [56]. Some interesting studies focusing on PHB ability as an enzyme inducer and DOX use animal models, having a more holistic vision where the liver is a major contributor towards DOX metabolization [57]. On the other hand, there is evidence that, in vivo, the aldo/keto reductase system is inhibited by PHB [29]. A recent study worked under the premise that inhibition of aldo/keto reductases was achieved by PHB. In the presence of PHB, a significant reduction in the formation of DOXol was seen in cytosolic fractions of the heart and liver of rats. In vivo, after a 3-day pre-treatment with PHB, the cumulative amount of DOXol in the bile and urine of pre-treated animals were significantly reduced. Conversely, no significant changes in DOX and doxorubicinol aglycones levels were seen [29]. Nevertheless, the literature’s contradictory results need to be seen in the light that PHB is a drug that acutely inhibits aldo/keto reductases, but after longer treatments, it is undoubtedly an enzymatic inducer with a large impact on metabolite profiling, and in our paradigm, no major changes were seen regarding DOX-inflicted toxicity.

Then, we had the opportunity to test the most relevant metabolites of DOX on differentiated AC16 cells. Several key considerations must be taken into account: (1) Currently, there is no available information regarding whether the biotransformation of DOX or its metabolites persists during incubation in differentiated AC16 cells. For the purpose of this study, we will assume that the observed effects are attributable to the drug given to the cells; (2) DOXol is the most abundant metabolite of DOX after hepatic metabolism and it is likely the metabolite that the heart encounters most frequently through the bloodstream; (3) Equimolar concentrations of all metabolites were selected to provide a mechanistic and comparative perspective; and (4) Recognizing the pivotal role of mitochondria in cardiac homeostasis, a detailed examination was conducted to assess the impact of the metabolites on mitochondrial membrane potential.

Among the metabolites analysed and according to the classical assays performed, DOXol was the most toxic metabolite. Its toxicity was significant at lower concentrations and the highest concentration tested (10 µM) was roughly at 48 h the toxic dose 50 (TD50) in both the MTT reduction and NR uptake assays. Even so, the toxicity was much lower than that of DOX, which pending on the MTT reduction assay had its TD50 around 0.5–1 µM, meaning that DOX was 10 times more toxic. Some pharmacokinetic considerations need to be taken into consideration when analysing these data: DOXol is more hydrophilic and that adds difficulty to its entry into cardiac cells, so when distributed (or added exogenously as in the present study) it may cause less toxicity than the parent drug, DOX. Early in vitro data showed DOXol to be more toxic than DOX [11]. Nonetheless, genetically altered animals featuring decreased expression of enzymes that are responsible for DOXol metabolite formation showed that after DOX administration, circulating levels of DOXol decreased. In that same paradigm, the histological and echocardiography damage caused by DOX administration decreased after the expression of enzymes responsible for DOXol formation was decreased [21]. Another study illustrated that increasing human carbonyl reductase expression in the hearts of mice led to an earlier onset of cardiotoxicity and a lower survival rate following DOX administration [58]. Although these studies are instrumental in understanding the role of metabolism on DOX-induced cardiotoxicity, it is crucial to bear in mind the intricate metabolic pathway of DOX outlined in Fig. 1, featuring multiple routes. Alterations in one pathway may promptly influence the proportions of all the other metabolites.

Two other commercially available metabolites were tested, both aglycones derived directly from DOX. Interestingly, they were less toxic than DOXol even though they are more hydrophobic than the parental drug and DOXol. In several studies, mitochondria have been seen as the major off-targets studied regarding aglycones toxicity [59–61], being that cardiac mitochondria seem to be significantly more sensitive to these metabolites [59]. In fact, in isolated mitochondria, a significant release of cytochrome *c* and a small decrease in the mitochondrial transmembrane potential were seen after incubation with aglycones [61]. In the present study, nonetheless, the parent drug, DOX, caused mitochondrial depolarization at the lower concentration tested (at 24 h incubation) whereas only double the concentration of the aglycones caused mitochondrial depolarization in the same model and time point. Moreover, in our model, the effect seen on mitochondrial membrane depolarization by aglycones was similar to DOXol, not corroborating mitochondria as their main target.

To sum up, this in vitro study aimed to investigate the role of DOX metabolism in its cytotoxicity towards differentiated AC16 cells. The modulation of DOX metabolism did not significantly affect its cytotoxicity. DOX’s chemical proprieties (e.g. amphiphilic nature of DOX) make passive membrane diffusion one of the key-mechanisms for its cellular transport, whereas the higher hydrophilic nature of DOXol makes it more prone to accumulate on the cells where the metabolism occurs and simultaneously makes it harder for it to cross membranes when in circulation [5, 6]. Those conditions (combined with a higher accumulation of DOX on mitochondria thanks to its high affinity to cardiolipin) would explain both the higher cytotoxicity and mitochondrial targeting of DOX. Nevertheless, these assumptions are challenged by the aglycones under study. Both have the secondary alcohol as DOXol but lack the sugar moiety, cleaved off during metabolism. That cleavage increases their lipophilicity and in theory increases the probability of cell entrance. Moreover, previous works [60, 62] on isolated mitochondria show their great potential to cause mitochondriopathy. However, our work clearly shows that these aglycones are in fact less toxic to cells and in particular mitochondria than DOX (and DOXol for that matter), and those mechanisms need to be further investgated. The metabolites, in our study, themselves exhibited some level of toxicity and considering the potential for both DOX and its metabolites to accumulate in the heart, it is important to address that they may contribute to a synergistic or additive effect on DOX's cardiotoxicity. In fact, DOX and DOXol accumulate for years on the heart, but if they can be converted to a safer cardiac metabolite that could potentially decrease chronic cardiotoxicity that could be important on a context of cardio-oncology. Yet, based on our observations, the process of metabolization does not seem to play a critical role in the cardiotoxicity of DOX.

## Data Availability

No datasets were generated or analysed during the current study.

## Abbreviations

7-DeoxyDOX: 7-Deoxydoxorubicin aglycone
1-ABT: 1-Aminobenzotriazole
CYP: Cytochrome
DAS: Diallyl sulphide
DMSO: Dimethyl sulphoxide
DMEM/F12: Dulbecco’s Modified Eagle’s Medium/Nutrient F-12 Ham
DOX: Doxorubicin
DOXol: Doxorubicinol
DOXone: Doxorubicinone
FBS: Foetal bovine serum
HS: Horse serum
MTP: Metyrapone
MTT: 3-(4,5-Dimethylthiazol-2-yl)-2,5-diphenyl tetrazolium bromide
NR: Neutral red
PBS: Phosphate buffer solution
PHB: Phenobarbital
SD: Standard deviation
3-MA: 3-Methyladenine
